# The intersection of travel burdens and financial hardship in cancer care: a scoping review

**DOI:** 10.1093/jncics/pkae093

**Published:** 2024-10-03

**Authors:** Arrianna Marie Planey, Lisa P Spees, Caitlin B Biddell, Austin Waters, Emily P Jones, Hillary K Hecht, Donald Rosenstein, Stephanie B Wheeler

**Affiliations:** Department of Health Policy and Management, Gillings School of Global Public Health, University of North Carolina at Chapel Hill, Chapel Hill, NC 27599-7411, United States; Lineberger Comprehensive Cancer Center, University of North Carolina at Chapel Hill, Chapel Hill, NC 27599, United States; Cecil G. Sheps Center for Health Services Research, University of North Carolina at Chapel Hill, Chapel Hill, NC 27516, United States; Lineberger Comprehensive Cancer Center, University of North Carolina at Chapel Hill, Chapel Hill, NC 27599, United States; Division of Pharmaceutical Outcomes and Policy, Eshelman School of Pharmacy, University of North Carolina at Chapel Hill, Chapel Hill, NC 27516, United States; Department of Health Policy and Management, Gillings School of Global Public Health, University of North Carolina at Chapel Hill, Chapel Hill, NC 27599-7411, United States; Lineberger Comprehensive Cancer Center, University of North Carolina at Chapel Hill, Chapel Hill, NC 27599, United States; Department of Health Policy and Management, Gillings School of Global Public Health, University of North Carolina at Chapel Hill, Chapel Hill, NC 27599-7411, United States; Lineberger Comprehensive Cancer Center, University of North Carolina at Chapel Hill, Chapel Hill, NC 27599, United States; Health Sciences Library, University of North Carolina at Chapel Hill, Chapel Hill, NC 27599, United States; Department of Health Policy and Management, Gillings School of Global Public Health, University of North Carolina at Chapel Hill, Chapel Hill, NC 27599-7411, United States; Lineberger Comprehensive Cancer Center, University of North Carolina at Chapel Hill, Chapel Hill, NC 27599, United States; Department of Psychiatry, School of Medicine, University of North Carolina at Chapel Hill, Chapel Hill, NC 27514, United States; Department of Hematology, School of Medicine, University of North Carolina at Chapel Hill, Chapel Hill, NC 27514, United States; Department of Health Policy and Management, Gillings School of Global Public Health, University of North Carolina at Chapel Hill, Chapel Hill, NC 27599-7411, United States; Lineberger Comprehensive Cancer Center, University of North Carolina at Chapel Hill, Chapel Hill, NC 27599, United States

## Abstract

**Background:**

In addition to greater delays in cancer screening and greater financial hardship, rural-dwelling cancer patients experience greater costs associated with accessing cancer care, including higher cumulative travel costs. This study aimed to identify and synthesize peer-reviewed research on the cumulative and overlapping costs associated with care access and utilization.

**Methods:**

A scoping review was conducted to identify relevant studies published after 1995 by searching 5 electronic databases: PubMed, Scopus, Cumulative Index of Nursing and Allied Health Literature (CINAHL), PsycInfo, and Healthcare Administration. Eligibility was determined using the PEO (Population, Exposure, and Outcomes) method, with clearly defined populations (cancer patients), exposures (financial hardship, toxicity, or distress; travel-related burdens), and outcomes (treatment access, treatment outcomes, health-related quality of life, and survival/mortality). Study characteristics, methods, and findings were extracted and summarized.

**Results:**

Database searches yielded 6439 results, of which 3366 were unique citations. Of those, 141 were eligible for full-text review, and 98 studies at the intersection of cancer-related travel burdens and financial hardship were included. Five themes emerged as we extracted from the full texts of the included articles: 1) Cancer treatment choices, 2) Receipt of guideline-concordant care, 3) Cancer treatment outcomes, 4) Health-related quality of life, and 5) Propensity to participate in clinical trials.

**Conclusions:**

This scoping review identifies and summarizes available research at the intersection of cancer care-related travel burdens and financial hardship. This review will inform the development of future interventions aimed at reducing the negative effects of cancer-care related costs on patient outcomes and quality of life.

## Introduction

It is estimated that at least a third of cancer patients in the United States have incurred higher-than-expected costs associated with cancer care, leading to financial distress or hardship (1). Financial toxicity (FT), defined as “patient-level impacts of the costs of cancer care” (2), is independently associated with increased mortality among cancer patients (3). In the robust body of research on FT (1), the costs of cancer care have been defined in holistic ways (4), including compounding monetary costs, from direct health-care expenses, and indirect costs such as childcare (5), accommodation, gas, transit, and parking fees (6). Nonmonetary and indirect costs of care include opportunity costs imposed through taking time off from work for appointments. Because affordability is a dimension of access to care, financial hardship can therefore be situated within the body of work on access to care (7).

Travel burdens are the cumulative temporal and financial costs of travel associated with receiving cancer treatment, which can exacerbate the stressors borne by patients. Travel burdens may exacerbate disparities in the timeliness of cancer screening and diagnosis, which are partly attributable to inequities in spatial access to screening services (7,8). Disparities in timely cancer screening and diagnosis also contribute to disparities across the continuum in cancer stage at diagnosis (8,9) and thereafter time-to-treatment (10,11), prognosis (12,13), and survival (14). In the case of colon cancer, variation in the timing of cancer diagnosis and tumor presentation at diagnosis explained Black/White disparities in survival to a greater degree than differences in treatment (12). Prior research has shown that, among [US] Medicare beneficiaries in the initial, survivorship, and end-of-life phases of cancer care, travel times in excess of an hour are associated with higher health-care costs (including out-of-pocket costs) and increased risk of hospitalization (15).

Disparities in spatial access to cancer care cut across sociodemographic categories and shape both site of care and treatment choices (16). As the share of the US population in survivorship now exceeds 18 million (17,18), there is a need for greater attention to the cumulative effects of burdensome travel on health-related quality of life (HRQoL) among cancer survivors (19). Patients in the United States face longer travel distances to access chemotherapy vs radiotherapy, and those who face barriers to transportation access are more likely to forgo needed care (20). Among breast cancer patients, longer travel distances are associated with higher likelihood of choosing mastectomy over breast-conserving surgery (21,22) and, subsequently, lower likelihood of post-mastectomy radiation therapy (23). Cancer care-related travel burdens are also associated with worse survival outcomes among cancer patients (24). Despite this, the results of a discrete choice experiment among patients with gynecologic cancers showed that 81% (50 out of 62) of participants required a 5-year survival benefit equal to or greater than 6% to justify 50 miles of additional travel to access a more specialized cancer care facility (25). The remaining 19% of participants expressed a preference to avoid additional travel for cancer care (25).

Cancer-care related travel burdens are inequitably distributed, with highest rates of transportation insecurity among impoverished people, people with lower levels of educational attainment, and people of color (26). Thus, these groups are vulnerable to disruptions in care due to lack of transportation (27). Rural-dwelling patients (28), particularly American Indian and low-income patients (29), experience the heaviest travel burdens for accessing cancer care in the United States (30,31). An estimated 20% of rural residents live further than 60 miles from the nearest medical oncologist (32). Evidence suggests that rural practices are slower to adopt newly approved immunotherapies (33), meaning that rural-dwelling patients may have to travel further to access the current standard treatments for their cancers. In the existing literature on travel burdens and cancer care utilization, the findings point to complex associations for timely receipt of guideline-concordant care (34), patient-reported outcomes (35), and survival (21), particularly at the intersection of race and rurality (36-39). Moreover, compared with their urban counterparts, rural-dwelling colorectal cancer survivors were twice as likely to report treatment-related financial hardship and twice as likely to be nonadherent with surveillance colonoscopy guidelines (40).

However, less is known about the intersection of cancer treatment-related travel burdens and financial hardship. Therefore, in this scoping review, we ask, “In the existing literature on cancer-related financial hardship, what is known about the role of travel burdens for care?”

## Materials and methods

We focused on synthesizing peer-reviewed literature on financial hardship and travel burdens borne by cancer patients and survivors. All included studies were published in English after 1995.

### Search strategy

In collaboration with a health sciences librarian, we iteratively developed our search strategy for the following databases: PubMed (National Library of Medicine, National Institutes of Health), Scopus (Elsevier), Cumulative Index of Nursing and Allied Health Literature (CINAHL) Plus with Full-Text (EBSCOhost), PsycInfo (EBSCOhost), and Healthcare Administration Database (ProQuest). The search strategy, conducted in English, was optimized to capture studies with key terms relating to “cancer,” “transportation,” “access,” and relevant cancer-related outcomes (such as survival, quality of life, hospitalization, and surveillance screening). Databases were searched from date of inception through the date the searches were executed, December 2, 2022, and results were limited to those in English language only. Full search strategies for all database searches are available in the Supplementary Tables S1-S5 (available online).

### Eligibility criteria

Eligibility was defined using the PEO (Population, Exposure, and Outcomes) method. Thus, our research criteria included clearly defined populations (cancer patients), exposures (financial hardship, toxicity, or distress; transportation and travel-related burdens), and outcomes (access to treatment, treatment outcomes, health-related quality of life, survival, and mortality). The inclusion and exclusion criteria are listed in Table 1.

**Table 1.** Summary table of inclusion and exclusion criteria

Inclusion criteria	Exclusion criteria
Focus on cancer patients or survivors	Does not focus on cancer patients or survivors eg, focus on cancer screening (pre-diagnosis)
Addresses travel burdens for cancer-related care (may use the terms “access to care,” “travel distance,” “travel burdens”)	Not specific to cancer-related care
Addresses the financial costs of cancer care (may use actual costs or conceptualize broadly as “financial toxicity,” “financial distress,” “financial hardship,” or “financial burden”)	Does not address the financial costs of cancer care
Focus on cancer patients’ or survivors’ health-care access and use	Does not address health-care access and utilization for cancer patients or survivors
Published in English language	Not written in English language
Focuses on care in the United States	Focus outside of United States

### Screening of abstracts and full text records

Citations identified by the search strategies were imported into Endnote, where they were deduplicated. All unique citations were imported into Covidence, where they underwent a 2-stage screening process by a minimum of 2 independent, blinded members of the study team. Records were first assessed by title and abstract, and any potentially meeting inclusion were assessed further using the full-text article. Conflicts during title and abstract and full-text screening were resolved by a third reviewer or team consensus. Data were extracted from all included articles, isolating study design, population, sample size, treatment modalities, demographic characteristics, study catchment area, key findings, and the definitions of rurality, financial burdens, and travel burdens. These data were synthesized across all included studies.

## Results

**Figure 1.**
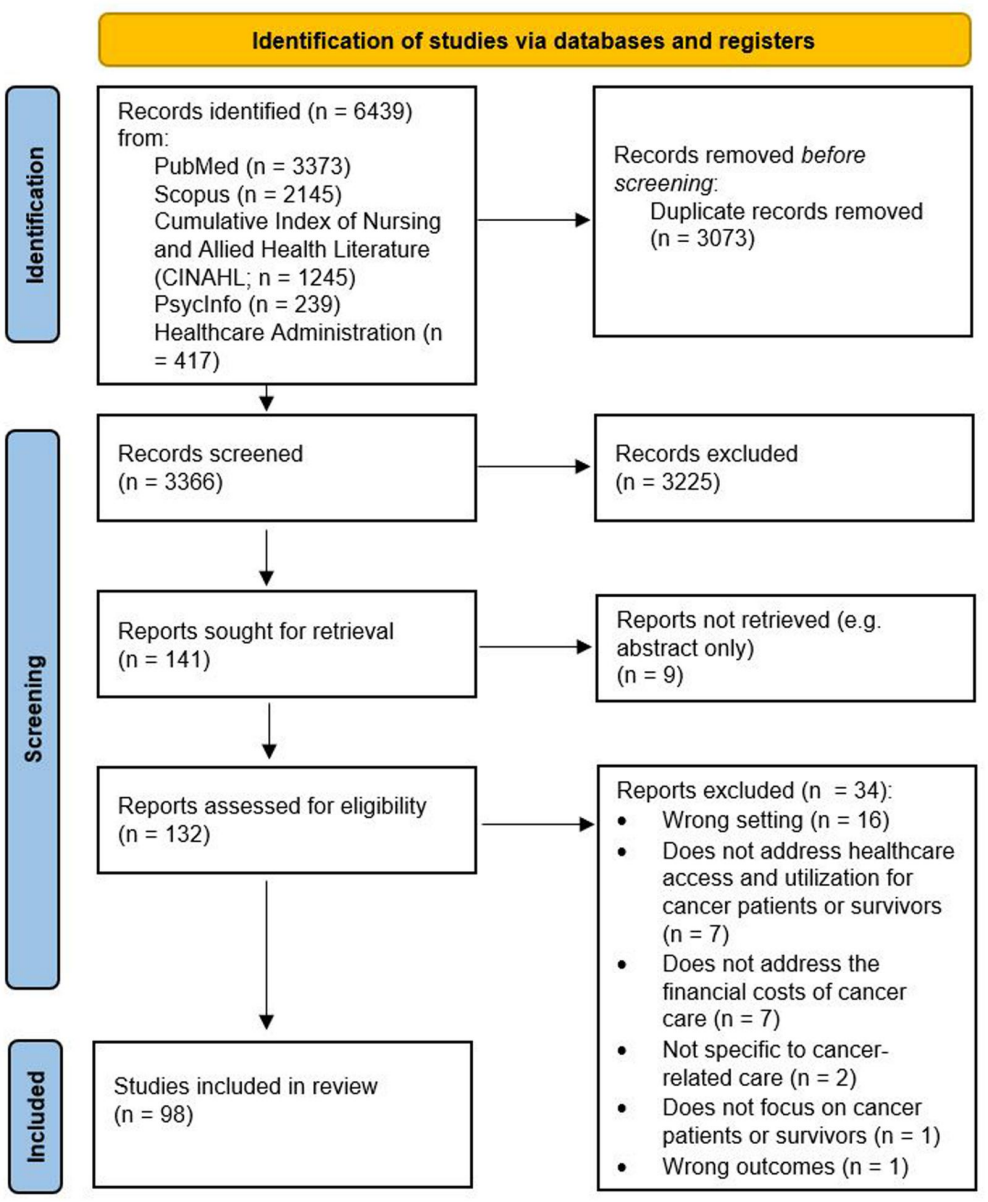
PRISMA flow diagram. A Preferred Reporting Items for Systematic Reviews and Meta-Analyses (PRISMA) flow diagram detailing the number of records in the initial database searches, the number of records whose abstracts we reviewed, and, thereafter, the number of full-text records that were screened and synthesized for this review.

Database searches yielded 6439 records, of which 3073 were duplicates. After de-duplication, 3366 unique records remained. Paired reviewers completed title and abstract screening for all unique records, and 3225 did not meet inclusion criteria and were excluded. Thereafter, we retrieved the full texts for 141 records, after which 9 were excluded because the full texts were not available or nonexistent (eg, published conference abstracts published as proceedings; see the Preferred Reported Items for Systematic Reviews and Meta-Analyses (PRISMA) chart in Figure 1). During our review of the 132 full-text articles, an additional 34 were excluded because they focused outside the United States or lacked a specific focus on financial hardship, travel burden, or cancer-related care. Therefore, the final number of included articles was 98 published between 1995 and 2022. No additional records were identified after searching the reference lists of the included articles. The inter-rater agreement for the full-text review stage was moderate, with agreement ranging from 0.519 to 0.711 (Cohen’s kappa = 0.356). However, this should be interpreted with caution, as Cohen’s kappa statistic is sensitive to the number of articles being reviewed, with higher values for larger samples (41). The PRISMA (42) flow diagram shows study selection process and is presented in Figure 1.

Of the 98 included articles, 66 (67.3%) were quantitative studies, 26 (26.5%) were qualitative studies, and 6 (6.1%) employed mixed methods (Table 2). The number of study participants varied between study types, ranging from 13 to 27 426 for quantitative studies and from 20 to 86 for mixed-methods studies. Among the included studies, the most common study methodology was cross-sectional analysis of survey data.

**Table 2.** Characteristics of included studies

Characteristics of included studies (n = 98)			
Study type	Quantitative (n = 66)	Qualitative (n = 26)	Mixed-methods (n = 6)
Number of study participants (range)	13–27 426	9–511	20–486
Patient ages	0–100	27–85	0–65
Study population	Cancer patients (64) Caregivers (1) Health care workers (1)	Cancer patients (17) Caregivers (6) Patient navigators (3) Community-based organization leaders (1) Health care workers (2) Cancer support agency staff (1)	Cancer patients (6)
Study designs	Experimental designs Randomized Controlled Trials (RCTs; 2) Discrete choice experiment (2) Evaluation studies Implementation Evaluations (1) Retrospective cohort studies Retrospective cohort study (2) Retrospective chart review (2) Case review (2)	Implementation of Patient Navigator program (1)	Intervention evaluation (2) Mixed-methods analysis of cancer-related GoFundMe campaigns (2)
Outcome variables	Financial toxicity (8) Out-of-pocket (OOP) costs (7) Psychological distress (1) Cancer treatment access (3) Treatment delays (3) Treatment discontinuation (1) Length of inpatient stays (1) Hospital readmissions (2) Receipt of follow-up care (1) Patient preferences for surveillance care (1) Loss to follow-up (1) Choice of treatment modality (12) Patient satisfaction Unmet social needs (5) Survival (5) HRQoL (5) Propensity to participate in clinical trials (13)	Financial distress (4) Psychological distress (1) Physical well-being (1) Ability to remain in the workforce (1) Barriers to cancer care receipt (6) Delays in care coordination (1) Receipt of surveillance screening Patient preferences for financial navigation (FN) programs (1) FN program staff perceptions of patient financial distress (2)	Cancer care receipt and enabling social supports (1) Patient navigation outcomes (1) Cancer-related crowdsourcing campaign outcomes (2)
Travel burden measures	Patient-reported travel-related costs (9) Patient-reported unmet transportation needs (4) Estimated monetary cost (mileage, frequency of trips, parking, lodging, parking, and meals) (8) Median travel distances/times (1) Round-trip travel times plus wait times (1) Distance from patient residence to hospital (18) One-way travel >1 hour (7)	Psychosocial burden of cancer-related travel (1) Distance to provider/facility (3) Barriers to travel due to timing of appointments (1) Logistical challenges of coordinating transportation (6) Aggregate travel costs, inclusive of lodging and food (6) Lack of comprehensive public transit (1)	Distance to provider/facility Travel duration (inclusive of layovers) Patient-reported transportation barriers

Study populations included cancer patients, caregivers, health-care workers, patient navigators, and cancer support staff. Among the included studies, 81.6% (80/98) focused on adult patient populations, whereas 4.1% (4/98) focused on child or adolescent patient populations, and 3.1% (3/98) of studies focused on both adult and pediatric patient populations. The remaining 11.2% (11/98) of studies did not report patient ages, in part because they were studies of patient or financial navigators who provide services to cancer patients.

### Synthesis of results, organized topically

Our results are organized across 5 themes that emerged as we extracted from the full texts of the included articles: (1) Cancer treatment choices, (2) Receipt of guideline-concordant care, (2) Cancer treatment outcomes, (4) Health-related quality of life (HRQoL), and (5) Propensity to participate in clinical trials. The synthesis that follows is generally organized according to the Population, Exposure, and Outcomes (PEO) framework. Throughout this section, we will use the term “financial hardship,” which is inclusive of FT (which is composed of material burden/out-of-pocket (OOP) costs, financial distress and behavioral responses to high cost burden) (43).

### Synthetic conceptual framework

Overall, the findings show that travel burdens and financial hardship can be considered compounding burdens throughout cancer survivors’ experience of care, resulting in cost-coping behaviors (15) that then affect receipt of guideline-concordant care, cancer treatment choices, and outcomes (eg, radiation treatment interruption) (44). Downstream of these factors are outcomes such as worsened health-related quality of life (45), increased symptom burden, and increased risk of mortality.

**Figure 2.**
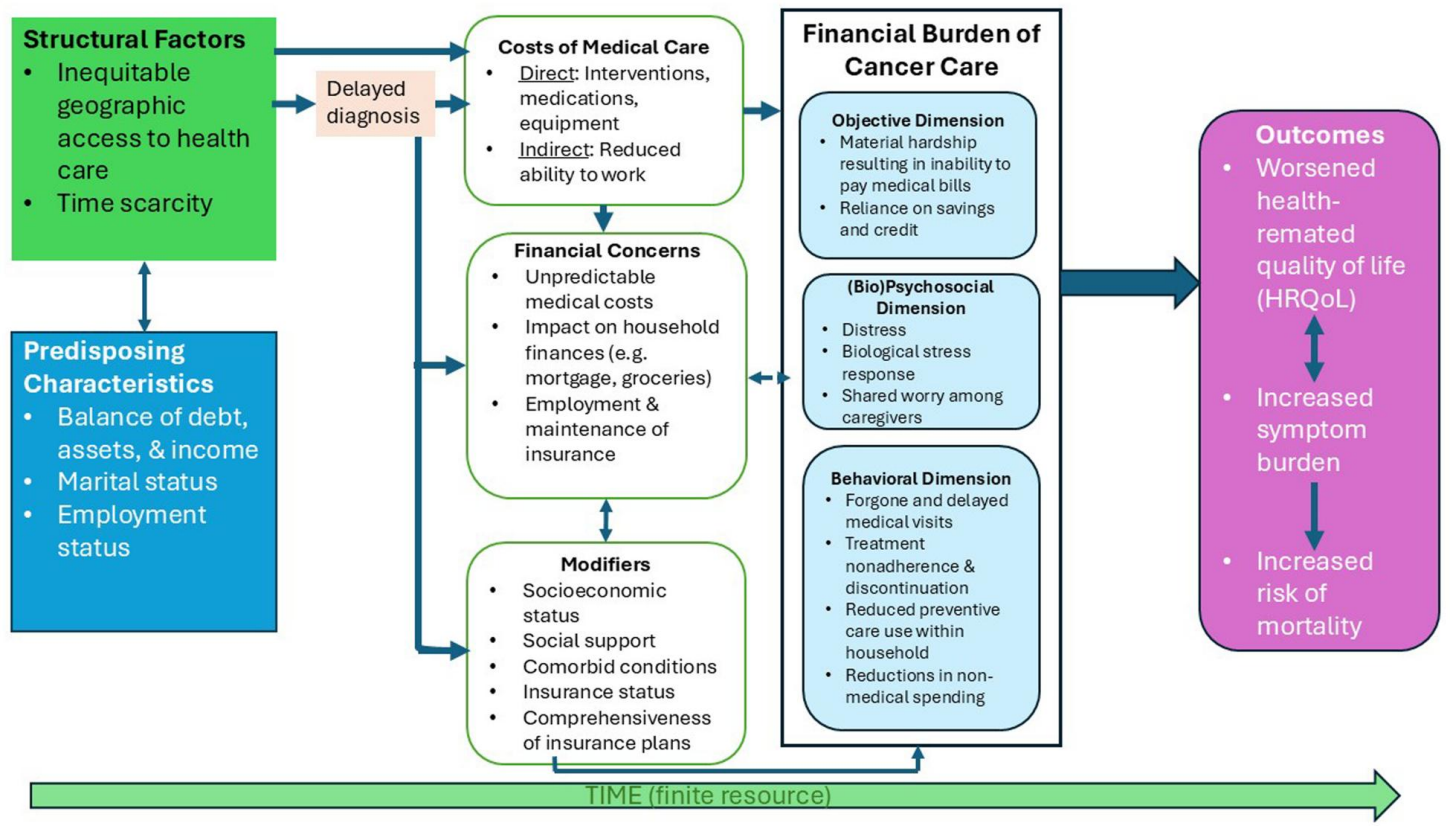
Conceptual framework linking cancer screening and care access inequities with financial burdens. A conceptual model representing potential relationships between inequities in cancer-related care access before and after diagnosis, and the financial burden of cancer care, with downstream outcomes that include worsened health-related quality of life (HRQoL), increased symptom burden, and increased risk of mortality. In this model, we introduce stress pathways (the biopsychosocial dimensions of financial burden or hardship) as a link between these dual burdens and worse patient outcomes among cancer survivors.

We summarize key findings in a conceptual model (Figure 2). This figure is an adaptation of previous conceptual models (46,47) linking cancer-related financial burdens with outcomes among cancer survivors. Consistent with Altice and colleagues (43), we incorporate the material hardships, psychological dimensions, and cost-coping dimensions of financial distress, but extend them to account for the bio-psychosocial facets that link financial distress with adverse outcomes via a stress pathway.

The adapted model includes structural and predisposing characteristics that influence the accessibility and affordability of care in the pre-diagnosis period. These factors include inequitable geographic access to health-care services at the axes of race, ethnicity, disability status, rurality, and socioeconomic status (especially in relation to timely receipt of cancer screening) (48-52). Specifically, we highlight access disparities pre- and post-diagnosis in this adapted model. For the purpose of this study, access is defined as a multidimensional concept, which includes the 5 “As of Access” (53): *Availability* (adequacy of the supply of health care workers relative to need), *Accessibility* (distance to care; other system-level barriers to or facilitators of help-seeking and service use), *Accommodation* (the degree to which a health-care system enables patient entry into health care; eg, walk-in appointment hours, or appointment slots outside of working hours), *Affordability* (including financing, reimbursements, and patient costs associated with help-seeking and service use), and *Acceptability* (degree of agreement between patient and health care worker expectations of and attitudes toward care) (7).

We note inequities in care access in the pre-diagnosis period as a precursor for delayed diagnoses because the latter is associated with increased intensity (and thus costs) of cancer care (15,54), which could mean greater financial burdens and cumulative travel burdens. In terms of predisposing characteristics, the model includes individual-level or household-level balance of debts, assets, and income, marital status, and employment status before diagnosis. Employment status is closely related to the modifiers of the relationship between financial concerns and the financial burdens of cancer care, principally through its relationship with socioeconomic status and insured status (particularly in the United States, where health-care insurance is often accessed through one’s employer) (55).

Prior research has shown that financial burdens or distress are associated with increased symptom burden (56) and increased risk of premature mortality among cancer survivors (57). Moreover, due to the dyadic nature of the caregiving relationship, financial distress affects the HRQoL for both cancer survivors (45,58,59) and caregivers (60).

### Compounding burdens: travel burdens and financial distress

This section synthesizes studies describing travel burdens and financial hardship to the exclusion of other cancer-related outcomes. Due to their compounding nature, travel burdens and financial hardship may jointly induce cost-coping behaviors among cancer survivors (43). A 2019 analysis of the National Cancer Institute’s Health Information and National Trends dataset estimated that approximately half (50.5%) of rural cancer survivors reported financial problems due to cancer compared with 38.8% of urban survivors (61).

Transportation comprises one of the biggest direct nonmedical costs of cancer care (62,63). Per a 2022 analysis of a trial for an out-of-pocket cost tracker, nonmedical costs (ie, transportation) represented the majority of cancer patients’ monthly out-of-pocket costs (mean $213, range $0-$587) (64). Moreover, given known racial disparities in wages and access to care, travel burdens and financial hardship may be considered compounding costs of accessing cancer care among patients of color. In a survey of nonmetastatic breast cancer survivors, Black patients were more likely to endorse transportation costs and the direct costs of health care as barriers to follow-up care (65). In a study of Black breast cancer patients in the Cleveland, Ohio metro area, respondents endorsed both transportation burdens and financial distress as reasons for their increased time-to-treatment (66).

Barriers may be higher still for patients who rely on public transportation to get to and from their appointments. In the United States, Black, Latinx, and people with lower incomes account for a disproportionate share of public transportation users (67,68). In the general population, unstable access to transportation interacts with poverty to reduce treatment and medication adherence (69). Within the Atlanta metropolitan region, cancer patients who use public transportation have longer travel times to access radiotherapy facilities (56 minutes, compared with 8 minutes for those who drove a personal vehicle), with the greatest disparities borne by Black cancer patients (70). Moreover, public transportation users may experience long waits outdoors that cause them to bear great discomfort. In a focus group study of Black women with breast cancer residing in Memphis, Tennesee, participants undergoing radiation therapy reported having to wait in the sun despite being advised against sun exposure after treatment (71).

It is important to note that travel distances are not a proxy for rurality. In a study of patient financial navigation program staff across 29 cancer centers in 7 US states, the respondents noted that urban-dwelling cancer survivors were more likely to lack a personal vehicle, which constrained survivors’ mobility, whereas rural-dwelling patients were more likely to incur additional costs associated with temporarily relocating to be near their cancer center (72). In another study, patients with advanced hematologic cancers and greater travel distances to access hematopoietic cell transplantation (HCT)—including the need to relocate to improve care access—were associated with greater financial distress (73). Relatedly, rural-dwelling patients with head and neck cancers who travel further for treatment have comparatively better outcomes than urban-dwelling patients residing within 5 miles of the facility (74). Thus, studies that do not disaggregate by rurality or urbanicity may have null findings for rural patients, who generally travel further to access cancer care and may experience better outcomes despite the added costs associated with care access.

### Cancer treatment choices

#### Site of care

The intersection of travel burdens and financial distress can alter patient behavior, including where one receives cancer care. This includes increased risk of hospitalization and greater likelihood of receiving care at a local hospital rather than a specialized hospital (15). Greater travel burdens for cancer care are associated with increased risk of emergency department use and hospital readmission among cancer patients who recently received major cancer surgeries (such as cystectomies, esophagectomies, lung resections, and pancreatectomies) (75). Notably, readmissions are associated with greater financial burdens, especially among patients who had poorer access to and use of postoperative care (76).

In a nationally representative survey evaluating patient willingness to travel to receive complex cancer surgeries at specialist hospitals, lower-income respondents (ie, those with incomes <$25 000) were the least willing to travel further to receive care at a specialty hospital. In this survey of willingness to travel for complex cancer surgeries, approximately 74% of respondents reported at least one barrier to travel, among which financial concerns were most common (77). Specific financial concerns included the possibility that the specialty hospital would be out-of-network and that the surgery would entail large out-of-pocket costs. Nearly all (94%) of respondents who reported barriers to travel for cancer surgery endorsed that they would be willing to travel if the costs of transportation, parking, and hotel stays were covered (77). For choice of cancer care site, financial and travel-related burdens continue into survivorship care, frequently provided by multiple specialties in heterogenous settings in the absence of a coordinated system (18). In one study, participants discussed costs associated with primary care access and use during survivorship, expressing concerns about continued and compounding financial and travel burdens (78).

Telehealth, defined as the use of telecommunication to enable remote health care service delivery (79), is a promising mode of care delivery that can reduce travel-related costs and travel-time (80,81). In one trial within the Veterans Affairs Health Care (VA) system, patients assigned to a video visit arm (vs an in-person visit arm) had significantly lower medical costs and lower indirect costs associated with cancer care, including the monetary costs of travel, distance traveled to access cancer care (median 0 vs 95 miles), travel time (0 vs 95 minutes), and workdays missed (0 vs 1 day). Among patients with head and neck cancers, telehealth follow-up consultations were estimated to reduce average (per patient) travel costs by approximately 28 hours traveling time and $900 (82). Unfortunately, disparities in access to telehealth persist for rural-dwelling cancer patients in the US South region (83), who have some of the heaviest travel burdens for cancer-related care (30).

#### Receipt of guideline-concordant care

In this section, we synthesize the research on the influence of travel and financial burdens on patient choice of cancer treatment modality (including stated and revealed preferences for surgical treatment) and receipt of follow-up care and surveillance screening in survivorship. The joint stressors of travel burdens and financial burdens for cancer care may also reduce cancer survivors’ receipt of guideline-concordant care. These outcomes include lower receipt of adjuvant chemotherapy within 90 days of colectomy (among colon cancer patients) (84), lower likelihood of post-mastectomy radiation therapy (among breast cancer patients) (23), and lower likelihood of receiving radiation therapy, in tandem with surgical treatment (among rectal cancer patients) (85). These burdens fall unevenly within cancer patient populations. Despite the fact that surgical treatment (resection) for stage I-III rectal cancer increases the odds of survival, older (ages 65 and older) and publicly insured (Medicaid and Medicare) rectal cancer patients were less likely to receive resections (86,87). Moreover, prior research has documented lower rates of breast-conserving treatment among breast cancer patients who traveled further for care—especially those residing in the South (88), where travel burdens to receive specialized cancer treatment are the highest in the nation (30,31).

### Choice of cancer treatment modality

The intersection of travel burdens and financial burdens can influence patients’ choice of cancer treatment modality, regardless of what is recommended as the “gold standard” for their cancer. Depending on cancer type, longer travel distances have been associated with lower receipt of surgical treatment and radiotherapy (RT). The association between travel distances and receipt of surgical treatment and/or radiotherapy will also differ by cancer site and specialist type, because the availability of oncology specialists differs in terms of geographic distribution, with a strong urban bias (30).

Due to the episodic nature of RT treatment, the cumulative travel and financial burdens are key considerations for cancer patients. In a sample of American Indian cancer survivors, 78% reported that the duration of RT was important, and 24% expressed a willingness to travel 25 miles for a standard course of RT (89). When offered the choice of a shorter course of RT, willingness to travel further (up to 50 miles) was nearly 4 times higher among employed respondents vs unemployed respondents (odds ratio [OR] = 3.64, *P* = .04) (89). In the case of soft tissue sarcomas, RT is the recommended treatment, and yet patients who have greater travel burdens to access cancer care and those with low incomes have lower likelihood of neoadjuvant or adjuvant RT receipt (90,91). Similarly, pediatric patients with primary malignancies of the central nervous system who reside more than 200 miles from a facility were more likely to receive the standard of care—proton beam therapy—between 2004 and 2012 (92).

### Patient preference for surgical treatments

Due to compounding financial and travel burdens borne of the episodic nature of nonsurgical treatments for cancer, patients with heavier travel burdens may opt for single-visit surgical treatments over multi-visit radiotherapy or chemotherapy. Among patients with early-stage glottic cancers, the compounding indirect cost of radiation therapy was consistently higher than that for endoscopic incision, resulting in more missed work hours, longer travel times, and greater total travel distances (93). Moreover, among patients with renal cell carcinoma (RCC) with cT1a (clinically inapparent) tumors, traveling 30 miles or more was associated with higher probability of receiving minimally invasive surgery (MIS) over partial nephrectomy (PN), as was higher income (>$62 000) and commercial insurance coverage (94).

In one discrete choice experiment (DCE) to elicit participants’ stated preferences, the authors calculated and added a cost attribute to better understand patient preferences for surgical treatment vs radiotherapy for primary basal cell carcinoma (95). The authors conducted 2 parallel DCEs—one with the cost attributes (composed of cost estimates of recurrence, re-excision, travel time, surgical time, waiting time for surgical results, financial costs of associated care) and one without—and compared the results. Compared with the respondents to a DCE survey without a cost attribute, respondents to the DCE with cost attributes preferred a surgical treatment with a lower probability of recurrence, lower surgery time, lower waiting time, and no risk for a re-excision, but specified the additional preference for a treatment with lower travel burdens and financial costs (95). Interestingly, per a stated preference survey and choice-based conjoint analysis of patients with facial melanomas, those who chose surgical treatments tended to emphasize lower rates of local recurrence, rather than the financial and travel burdens associated with alternative treatment modalities (96).

### Receipt of follow-up care and surveillance screening in survivorship

Travel and financial burdens also influence the use of follow-up care and surveillance screening in survivorship. Among rural-dwelling cervical cancer survivors in North Carolina (97), barriers to surveillance screening (pap smears) included long travel times and high out-of-pocket costs. HPV-positive oropharyngeal cancer survivors, who tend to be more highly educated with higher socioeconomic status (98), have higher 5-year survival rates than their counterparts with HPV-negative head and neck cancers (>85% and <50%, respectively) (99). Due to their high rates of disease-free survival, patients with HPV-positive head and neck cancers are ideal candidates for surveillance screening in survivorship; however, patients with stage I or II HPV-related oropharyngeal cancers reported travel burdens, time off work, and nonmedical costs as barriers or burdens associated with surveillance visits (99). The survey respondents were undergoing the current standard of survivorship care, which entails quarterly clinical visits for nasopharyngolaryngoscopy during the first 2 years after treatment, thereafter reduced to every 4 to 6 months in years 3-5 (99).

### Cancer treatment outcomes

There are several pathways by which the intersection of financial burdens and travel burdens influence cancer treatment outcomes, including higher likelihood of care discontinuation and reductions in peri- and postoperative follow-up care receipt, which result in lower overall survival. In the section that follows, we synthesize the research on the influence of travel and financial burdens on patients’ likelihood of care discontinuation, reductions in perioperative and postoperative care receipt, and overall survival.

#### Overall survival

Overall survival can be considered to be downstream of the effects of cancer-related financial distress, including, but not limited to, higher risk of care discontinuation and increased symptom burden. Prior studies have identified differential mortality among patients treated at academic medical centers (AMCs) vs non-AMCs, despite greater distances traveled to access care at AMCs (100). In contrast, in a sample of prostate cancer patients, those who traveled 30 miles or more had a *reduced* risk of death (101). Notably, however, overall survival was highest among commercially insured patients and lowest among Medicare and Medicaid-insured patients, who were more likely to have delayed prostate cancer diagnosis (later stage at diagnosis) (101). Similarly, among lower-income patients who received surgical resection for extrahepatic biliary malignancies, greater travel burdens for cancer care are associated with lower survival (102).

Disparities in patients’ and families’ abilities to bear the financial and travel burdens associated with cancer care have downstream effects on overall survival. Among pediatric glioblastoma patients, disparities in the receipt of proton radiation therapy (PRT) persist, with lower receipt among rural-dwelling patients and Medicaid-insured patients and greater likelihood of receipt among White, higher-income, and urban-dwelling patients (103). There is also an inverse relationship between travel distance to pediatric glioblastoma treatment and survival, wherein patients who traveled less than 5 miles had the shortest overall survival (11.8 months), whereas those who traveled more than 50 miles had the longest survival (12.9 months) (104). Overall, financial strain associated with cancer care was more directly related to overall survival than travel times to access PRT. However, we note that these relationships are heterogeneous across cancer sites and types. In contrast with findings for patients with solid tumors, among acute myeloid leukemia (AML) patients receiving remission induction therapy, neither distance to care nor socioeconomic status were associated with overall survival (105).

#### Higher likelihood of care discontinuation

In key informant interviews with financial navigators at cancer centers in Kentucky, participants noted that high burdens of financial distress influenced patient decisions to delay or forgo care—a pattern possibly exacerbated by patients’ limited ability to engage in conversations about the costs of cancer care (106). Per a 2017 systematic review, the proportion of US cancer survivors experiencing financial distress who were nonadherent to their prescription medication due to cost ranged from 4% to 45% (43). Gynecologic cancer patients had higher risk of nonadherence-related treatment interruptions if they either self-reported inability to afford medical care (OR, 5.69; 95% Confidence Interval [CI] = 1.12 to 28.9; *P* = .036) or named a lack of transportation (OR, 20.5; 95% CI = 2.69 to 156.7; *P* = .004) (107). Moreover, patients who jointly reside in more impoverished neighborhoods and travel further for care are more likely to have unplanned care discontinuation (44).

#### Lower likelihood of perioperative and post-operative follow-up care

Among rural-dwelling breast cancer patients, greater distance from radiotherapy facilities was associated with a lower likelihood of opting for breast-conserving treatment, which requires radiation therapy after surgery (108). Another study found that there was a lower likelihood of perioperative chemotherapy receipt among patients with muscle-invasive bladder cancer (109). Longer travel distances and lack of insurance—important factors in care (un)affordability—were associated with lower receipt of cystectomy among patients with muscle-invasive bladder cancer (110). Moreover, among patients who received surgery for head and neck cancers, those who lived further away from the surgery-providing hospital were less likely to receive follow-up, postoperative radiation therapy (PORT) at that hospital (111).

### Health-related quality of life (HRQoL)

Both the financial burdens and the travel costs of cancer care affect cancer survivors’ quality of life. Defined as the physical and psychosocial effects downstream of the stressors of cancer diagnosis and treatment, HRQoL includes the mental health effects of cancer survivorship and treatment (112-114). Although patients who did not have chronic conditions before their cancer diagnosis tend to have better HRQoL, the development of chronic conditions during or after cancer treatment exacerbates declines in HRQoL among cancer survivors (112). Financial hardship and time scarcity (exacerbated by treatment-related travel) (115) may undermine cancer patients’ ability to engage in health-enabling activities, thus affecting their HRQoL (112). This may be compounded by the effects of cancer treatments on daily functioning, such as pain and limited mobility.

These effects include worsened mental health in the period after a cancer diagnosis, extending into survivorship (defined as the period between cancer diagnosis and end of life) (18). In one study, Hispanic women at the US–Mexico border region who experienced both long travel distances to care and financial burdens had elevated risk of depression in the year after a breast cancer diagnosis (116). These findings are consequential because worse HRQoL is associated with increased symptom burden (112). These effects can be observed among caregivers as well. Among Pacific Islander parents of pediatric cancer patients receiving care in Hawaii, inter-island relocations entailed leaving jobs and place-based social supports, and these parents reported distress that took the form of anxiety with somatic symptoms (eg, unexplainable fevers, loss of appetite, pain) (117).

#### Caregiving and social support

Social support is both a potential moderator or mediator of the relationship between travel burdens for cancer care and financial distress. Caregivers often provide transportation, and longer distances may be associated with greater indirect, travel-related costs for both patients and caregivers due to their dyadic relationship (118). Interestingly, one study showed that reported caregiver financial burden was higher among caregivers who lived closer to their care recipient. In a sample of 347 Black caregivers of cancer survivors, the mean financial burden score was 7.4 (with 3–5 representing “low” burden, 6–8 representing “medium” burden, and scores exceeding 9 representing “high” financial burden), caregivers who lived within 20 minutes of the care recipient had higher financial burden compared with caregivers who shared a household with a cancer survivor (7.8, 95% CI = 7.4 to 8.2 versus 7.3, 95% CI = 7.0 to 7.5) or lived further away from their care recipient (>20 minutes; (7.4, 95% CI = 6.7 to 8.1) (119).

In a cohort of parent caregivers of pediatric cancer patients, nearly 1 in 3 caregivers reported moving after their child’s diagnosis—a third of whom attributed their child’s cancer as the cause of their relocation (120). Rural-dwelling patients and caregivers had higher initial travel burdens for cancer care (exceeding an hour of travel time), resulting in more missed days of work. Additionally, rural-dwelling patients and caregivers had heavier overall travel and financial burdens associated with cancer care. Of the total sample, a third of parents reported quitting their jobs, suggesting reverberating financial consequences of childhood cancer. In a case report pairing chart review with semistructured interviews with Pacific Islander parents of pediatric cancer survivors, nearly half of parents relocated their immediate families from many islands to Hawaii to enable better access to cancer care in Honolulu and defray the costs of inter-island travel (117). Parents in this sample hailed from American Samoa, the Commonwealth of the Northern Marianas (CNMI), the Federated States of Micronesia (FSM), Guam, the Republic of the Marshall Islands, and Kiribati and traveled distances upward of 2000 miles to access cancer care (117). Notably, all of the island nations except Guam and American Samoa provided financial and housing support for relocation during cancer care.

In a qualitative study of barriers and facilitators to cancer treatment adherence among older Black cancer patients and their caregivers in rural North Carolina, the authors identified 3 themes: transportation-related barriers, financial barriers, and social assistance that facilitated adherence to cancer treatment protocols (121). Cancer survivors identified barriers relating to *Accommodation* (53), such as long wait times for appointments, lack of convenient appointment times, and long waits for transportation after appointments. In combination, these barriers prevented cancer survivors from coordinating transportation with caregivers.

Reflecting the dyadic nature of the caregiving relationship (118), the costs borne by caregivers of cancer survivors are substantial. For caregivers, transportation may dovetail with financial burdens through the spillover financial effects of out-of-pocket costs for gas, parking, and the additional indirect opportunity costs of taking time off work (121). Spillover effects for caregivers included missing bill payments for their own households (119). In a study of Black caregivers of cancer survivors in Detroit, nearly two-thirds of caregivers reported costs associated with caregiving, and 38% of respondents reported high degrees of financial hardship (119). The most commonly reported caregiving-related costs were transportation/logistics support, medications, and groceries, and approximately 16.5% of caregiver respondents reported assisting with paying medical bills (119). These findings are consistent with a previous review of the financial burden borne by caregivers of cancer survivors (60).

In the absence of caregiver support, patients may incur more transportation and lodging costs as they seek treatment. Among rural-dwelling women with breast cancer who were simultaneously experiencing intimate partner violence (IPV), financial and temporal constraints influenced their treatment modality and site of care (choosing radiation therapy at a closer location) (122). In a focus group study, breast cancer patients undergoing chemotherapy who did not have transportation support reported feeling unsafe while driving mountainous routes to home, in part due to their treatment side effects (123).

### Propensity to participate in clinical trials

Thirteen of the included studies addressed the influence of travel burdens on propensity to participate in clinical trials among cancer patients experiencing financial distress. This has major implications for cancer-related clinical trial accrual. Among participants in Phase I clinical trials, financial burdens were greatest for low-income cancer patients who have to travel farther to access cancer care (124).

Propensity to participate in clinical trials is influenced by multilevel factors, such as area-level socioeconomic status (125), patient age (126), patient medical mistrust (127), and patient awareness of state laws requiring insurance companies to reimburse costs associated with clinical trial participation (128). At the structural or health system level, availability of clinical trials differs by rurality. A 2022 study showed that breast and lung cancer patients at urban cancer centers were more likely to be treated at sites with available clinical trials for their cancer type, vs comparable patients at rural sites (3.56 times higher for urban-dwelling breast cancer patients and 4.27 times more likely for urban-dwelling lung cancer patients, respectively) (129). Furthermore, per an evaluation of breast cancer survivors’ stated reasons for declining to participate in a RCT for complementary/alternative medical treatments, the most frequent reasons for non-participation included logistical barriers associated with employment and coordinating childcare (33.3%) and transportation-related barriers (30.6%) (130).

For cancer survivors, the barriers to cancer care are also barriers to clinical trial participation. However, health system interventions such as cancer care equity programs (CCEP) can potentially increase clinical trial participation among cancer survivors with the greatest unmet need (131). In this instance, for patients with incomes less than 400% federal poverty level (FPL), the CCEP services included full reimbursement of patients’ travel and lodging expenses. Compared with patients whose income was greater than 400% FLP, the recipients of the CCEP services were more likely to report concerns about the financial burden of participating in the clinical trial (56% vs 11%), out-of-pocket medical costs (47% vs 14%), and travel-related costs (eg, transportation access; 69% vs 11%) and lodging (60% vs 9%) and adequate insurance coverage (43% vs 14%) (131). As a result of receiving financial assistance, cancer patients were more likely to participate in clinical trials (131). In a follow-up study, the authors quantified the financial burden of clinical trial participation, finding that patients who were eligible for a financial navigation intervention were more likely to report travel to medical appointments as their most significant financial concern (24.0% vs 7.0%, *P* = .001) and to report forgoing medical care because of worries about costs (14.2% vs 1.1%, *P* = .001), compared with the clinical trial participants who were ineligible for financial navigation (132). Over time, clinical trial participants who received financial navigation reported less concern about the cost of travel and lodging associated with clinical trial participation, but the associated financial burden of cancer care did not significantly change (132).

## Discussion

This scoping review synthesizes existing research on the overlap between financial hardship and travel burdens for cancer care. In considering travel burdens for accessing cancer care as added costs of care, this scoping review is situated at the intersection of travel burdens and financial burdens. Importantly, we identify thematic areas in this research: 1) Cancer treatment choices, 2) Receipt of guideline-concordant care, 3) Cancer treatment outcomes, 4) Health-related quality of life (HRQoL), and 5) Propensity to participate in clinical trials.

Prior research suggests that rural-dwelling cancer survivors experience heavier financial burdens associated with cancer care (61). Additionally, rural-dwelling cancer patients are likely to report being less satisfied with their ease of access to cancer care and less likely to report having their health care needs met at a single facility (133). This is partially reflective of the greater travel burdens borne by rural-dwelling cancer patients (30,31). For working-age cancer patients, the relationship between travel burdens and financial burdens may be modified by disparities in eligibility and access to paid sick leave, which fall along the axes of race, ethnicity, and rurality (134,135). Moreover, consistent with prior literature on travel-related costs as contributors to cancer cost burden (6,136), cancer patients who reported barriers to travel for cancer surgery expressed a willingness to travel if the costs of transportation, parking, and hotel stays were covered (77).

## Study limitations

The search strategies were limited to results in English language only, potentially removing relevant articles meeting inclusion written in other languages. Most of the included studies precede the wider adoption of molecular and immune therapies, which are more costly and pose even greater financial burdens for cancer patients (137). Because the studies in this review largely focused on radiotherapy, chemotherapy, and surgical treatments, they may collectively underestimate the degree of financial burden borne by patients receiving molecular and immune therapies.

### Future directions

A key opportunity for further research is understanding how cancer-related financial and travel burdens jointly affect members of patients’ households (54). This may be especially concerning amid the COVID pandemic, which has disproportionately affected cancer patients and survivors (138), with worse effects among Black cancer patients and survivors (139). With the end of the Public Health Emergency (PHE), the resumption of Medicaid redeterminations and disenrollments and subsequent coverage losses (140), and resulting catastrophic costs (141), and care disruptions may especially burden low-income and disabled cancer patients.

## Conclusion

When considering the totality of the cancer burden, the material out-of-pocket costs of care, alongside transportation-related time and monetary cost of travel for appointments, all contribute to the out-of-pocket obligation placed onto patients. These contribute to substantial distress and cost-coping behaviors that alter care. To alleviate the distress of going through major medical care, health systems can respond by making the process of accessing care easier. Policymakers, insurance payers, and patient navigators each hold authority to consolidate resources and prioritize the financial and logistic impact of accessing health care. Clinicians may incorporate practical scheduling and affordability as a realistic decision point in scheduling complex cancer care, while researchers can design trials and other studies in such a way to limit travel and cost burdens to more equitably reach people in need. As cancer treatment modalities evolve, practical considerations about equity in who can access treatment must remain at the forefront.

## Supplementary Material

pkae093_Supplementary_Data

## Data Availability

No new data were generated or analyzed in this review article. All search strategies are detailed in the Supplementary Methods.
