# Supplementary material for: The intersection of travel burdens and financial hardship in cancer care: a scoping review
Source: JNCI Cancer Spectr. 2024 Oct 3;8(5):pkae093. doi: 10.1093/jncics/pkae093 (PMC11519048; doi:10.1093/jncics/pkae093)
Supplement: pkae093_Supplementary_Data [file pkae093_supplementary_data.docx]

## **Supplementary Methods**

**Supplementary Table 1 - PubMed (National Library of Medicine/National Institutes of Health): Searched December 2, 2022**

| Search | Query | Results |
| --- | --- | --- |
| 1 | “Neoplasms”[MeSH terms] OR “Early Detection of Cancer”[MeSH terms] OR cancer[Title/Abstract] OR cancers[Title/Abstract] OR malignancy[Title/Abstract] OR malignancies[Title/Abstract] OR tumor[Title/Abstract] OR tumors[Title/Abstract] OR tumour[Title/Abstract] OR tumours[Title/Abstract] OR neoplasm[Title/Abstract] OR neoplasms[Title/Abstract] | 4797846 |
| 2 | “Transportation”[MeSH terms] OR “Transportation of Patients” [MeSH terms] OR transportation[Title/Abstract] OR “distance to care”[Title/Abstract] OR “travel distance”[Title/Abstract] OR ((transport[Title/Abstract] OR transports[Title/Abstract] OR transported[Title/Abstract] OR transporting[Title/Abstract] OR "Travel"[Mesh] OR travel[Title/Abstract] OR traveled[Title/Abstract] OR traveling[Title/Abstract] OR travelled[Title/Abstract] OR travelling[Title/Abstract] OR "Residence Characteristics"[Mesh] OR residential[Title/Abstract] OR neighborhood[Title/Abstract] OR distance[Title/Abstract] OR residence[Title/Abstract] OR rural[Title/Abstract] OR "Rural Population"[Mesh] OR rurality[Title/Abstract]) AND (patient[Title/Abstract] OR patients[Title/Abstract] OR inpatient[Title/Abstract] OR inpatients[Title/Abstract])) | 301397 |
| 3 | "financial toxicity"[Title/Abstract] OR "financial stress"[Title/Abstract] OR "financial distress"[Title/Abstract] OR "financial burden"[Title/Abstract] OR "financial hardship"[Title/Abstract] OR "financial hardships"[Title/Abstract] OR "health care cost"[Title/Abstract] OR "health care costs"[Title/Abstract] OR "healthcare cost"[Title/Abstract] OR "healthcare costs"[Title/Abstract] OR "Health Expenditures"[Mesh] OR expenditure[Title/Abstract] OR expenditures[Title/Abstract] OR income[Title/Abstract] OR "drug cost"[Title/Abstract] OR "drug costs"[Title/Abstract] OR cost[Title/Abstract] OR costs[Title/Abstract] OR finances[Title/Abstract] OR financial[Title/Abstract] OR "Cost of Illness"[Mesh] OR "cost of illness"[Title/Abstract] OR "insurance coverage"[Title/Abstract] OR out-of-pocket[Title/Abstract] OR "out of pocket"[Title/Abstract] OR "insurance premium"[Title/Abstract] OR "insurance premiums"[Title/Abstract] OR "insurance cost"[Title/Abstract] OR "insurance costs"[Title/Abstract] OR affordable[Title/Abstract] OR affordability[Title/Abstract] | 993244 |
| 4 | outcome[Title/Abstract] OR outcomes[Title/Abstract] OR survival[Title/Abstract] OR “quality of life”[Title/Abstract] OR quality-of-life[Title/Abstract] OR hospitalization[Title/Abstract] OR hospitalizations[Title/Abstract] OR emergency[Title/Abstract] OR emergencies[Title/Abstract] OR screening[Title/Abstract] OR diagnose[Title/Abstract] OR diagnosis[Title/Abstract] OR diagnoses[Title/Abstract] OR treatment[Title/Abstract] OR treatments[Title/Abstract] OR therapy[Title/Abstract] OR therapies[Title/Abstract] OR chemotherapy[Title/Abstract] OR chemotherapies[Title/Abstract] OR radiation[Title/Abstract] OR appointment[Title/Abstract] OR appointments[Title/Abstract] OR discontinue[Title/Abstract] OR discontinuation[Title/Abstract] OR delay[Title/Abstract] OR delays[Title/Abstract] OR cancel[Title/Abstract] OR cancels[Title/Abstract] OR cancellation[Title/Abstract] OR no-show[Title/Abstract] OR no-shows[Title/Abstract] OR employment[Title/Abstract] OR medication[Title/Abstract] OR death[Title/Abstract] OR deaths[Title/Abstract] OR fatality[Title/Abstract] OR fatalities[Title/Abstract] OR "Mortality"[Mesh] OR mortality[Title/Abstract] OR adherence[Title/Abstract] OR nonadherence[Title/Abstract] | 11330884 |
| 5 | #1 AND #2 AND #3 AND #4 | 2731 |
| 6 | meta analysis[Publication Type] OR systematic review[Publication Type] OR review[Publication Type] OR systematic-review[ti] OR meta-analysis[ti] OR scoping-review[ti] OR literature-review[ti] | 3291408 |
| 7 | #5 NOT #6 | 2474 |
| 8 | #7 AND (English[language]) | 2393 |

**Supplementary Table 2 - Scopus (Elsevier): Searched December 2, 2022**

| Search | Query | Results |
| --- | --- | --- |
| 1 | Title-ABS (cancer OR cancers OR malignancy OR malignancies OR tumor OR tumors OR tumour OR tumours OR neoplasm OR neoplasms) | 4010638 |
| 2 | TITLE-ABS (transportation OR "distance to care" OR "travel distance" OR ((transport OR transports OR transported OR transporting OR travel OR traveled OR traveling OR travelled OR travelling OR residential OR neighborhood OR distance OR residence OR rural OR rurality) AND (patient OR patients OR inpatient OR inpatients))) | 549816 |
| 3 | Title-ABS ("financial toxicity" OR "financial stress" OR "financial distress" OR "financial burden" OR "financial hardship" OR "financial hardships" OR "health care cost" OR "health care costs" OR "healthcare cost" OR "healthcare costs" OR expenditure OR expenditures OR income OR "drug cost" OR "drug costs" OR cost OR costs OR finances OR financial OR "cost of illness" OR "insurance coverage" OR out-of-pocket OR "out of pocket" OR "insurance premium" OR "insurance premiums" OR "insurance cost" OR "insurance costs" OR affordable OR affordability) | 382968 |
| 4 | Title-ABS (outcome OR outcomes OR survival OR "quality of life" OR quality-of-life OR hospitalization OR hospitalizations OR emergency OR emergencies OR screening OR diagnose OR diagnosis OR diagnoses OR treatment OR treatments OR therapy OR therapies OR chemotherapy OR chemotherapies OR radiation OR appointment OR appointments OR discontinue OR discontinuation OR delay OR delays OR cancel OR cancels OR cancellation OR no-show OR no-shows OR employment OR medication OR death OR deaths OR fatality OR fatalities OR mortality OR adherence OR nonadherence) | 17253006 |
| 5 | #1 AND #2 AND #3 AND #4 | 2616 |
| 6 | TITLE ("meta analysis" OR "systematic review" OR systematic-review OR meta-analysis OR scoping-review OR literature-review) | 413081 |
| 7 | #5 AND NOT #6 | 2557 |
| 8 | #7 AND ( EXCLUDE ( DOCTYPE , "re" ) OR EXCLUDE ( DOCTYPE , "cp" ) OR EXCLUDE ( DOCTYPE , "cr" ) ) | 2245 |
| 9 | #8 AND ( LIMIT-TO ( LANGUAGE , "English" ) ) | 2145 |

**Supplementary Table 3 - CINAHL (EBSCOhost) – Searched December 2, 2022**

| Search | Query | Results |
| --- | --- | --- |
| 1 | (MH "Neoplasms+") OR (MH "Early Detection of Cancer") OR (TI cancer OR AB cancer) OR (TI cancers OR AB cancers) OR (TI malignancy OR AB malignancy) OR (TI malignancies OR AB malignancies) OR (TI tumor OR AB tumor) OR (TI tumors OR AB tumors) OR (TI tumour OR AB tumour) OR (TI tumours OR AB tumours) OR (TI neoplasm OR AB neoplasm) OR (TI neoplasms OR AB neoplasms) | 846496 |
| 2 | (MH "Transportation+") OR (MH "Transportation of Patients+") OR TI transportation OR AB transportation) OR (TI "distance to care" OR AB "distance to care") OR (TI "travel distance" OR AB "travel distance") | 41335 |
| 3 | (((TI transport OR AB transport) OR (TI transports OR AB transports) OR (TI transported OR AB transported) OR (TI transporting OR AB transporting) OR (MH Travel+) OR (TI travel OR AB travel) OR (TI traveled OR AB traveled) OR (TI traveling OR AB traveling) OR (TI travelled OR AB travelled) OR (TI travelling OR AB travelling) OR (MH "Residence Characteristics+") OR (TI residential OR AB residential) OR (TI neighborhood OR AB neighborhood) OR (TI distance OR AB distance) OR (TI residence OR AB residence) OR (TI rural OR AB rural) OR (MH "Rural Population+") OR (TI rurality OR AB rurality)) AND ((TI patient OR AB patient) OR (TI patients OR AB patients) OR (TI inpatient OR AB inpatient) OR (TI inpatients OR AB inpatients))) | 70123 |
| 4 | #3 OR #4 | 107076 |
| 5 | (TI "financial toxicity" OR AB "financial toxicity") OR (TI "financial stress" OR AB "financial stress") OR (TI "financial distress" OR AB "financial distress") OR (TI "financial burden" OR AB "financial burden") OR (TI "financial hardship" OR AB "financial hardship") OR (TI "financial hardships" OR AB "financial hardships") OR (TI "health care cost" OR AB "health care cost") OR (TI "health care costs" OR AB "health care costs") OR (TI "healthcare cost" OR AB "healthcare cost") OR (TI "healthcare costs" OR AB "healthcare costs") OR (MH "Health Expenditures+") OR (TI expenditure OR AB expenditure) OR (TI expenditures OR AB expenditures) OR (TI income OR AB income) OR (TI "drug cost" OR AB "drug cost") OR (TI "drug costs" OR AB "drug costs") OR (TI cost OR AB cost) OR (TI costs OR AB costs) OR (TI finances OR AB finances) OR (TI financial OR AB financial) OR (MH "Cost of Illness+") OR (TI "cost of illness" OR AB "cost of illness") OR (TI "insurance coverage" OR AB "insurance coverage") OR (TI out-of-pocket OR AB out-of-pocket) OR (TI "out of pocket" OR AB "out of pocket") OR (TI "insurance premium" OR AB "insurance premium") OR (TI "insurance premiums" OR AB "insurance premiums") OR (TI "insurance cost" OR AB "insurance cost") OR (TI "insurance costs" OR AB "insurance costs") OR (TI affordable OR AB affordable) OR (TI affordability OR AB affordability) OR (MH affordability) | 337765 |
| 6 | (TI outcome OR AB outcome) OR (TI outcomes OR AB outcomes) OR (TI survival OR AB survival) OR (MH "Quality of Life+") OR (TI "quality of life" OR AB "quality of life") OR (TI quality-of-life OR AB quality-of-life) OR (MH "Hospitalization+") OR (TI hospitalization OR AB hospitalization) OR (TI hospitalizations OR AB hospitalizations) OR (TI emergency OR AB emergency) OR (TI emergencies OR AB emergencies) OR (TI screening OR AB screening) OR (TI diagnose OR AB diagnose) OR (TI diagnosis OR AB diagnosis) OR (TI diagnoses OR AB diagnoses) OR (TI treatment OR AB treatment) OR (TI treatments OR AB treatments) OR (TI therapy OR AB therapy) OR (TI therapies OR AB therapies) OR (TI chemotherapy OR AB chemotherapy) OR (TI chemotherapies OR AB chemotherapies) OR (TI radiation OR AB radiation) OR (TI appointment OR AB appointment) OR (TI appointments OR AB appointments) OR (TI discontinue OR AB discontinue) OR (TI discontinuation OR AB discontinuation) OR (TI delay OR AB delay) OR (TI delays OR AB delays) OR (TI cancel OR AB cancel) OR (TI cancels OR AB cancels) OR (TI cancellation OR AB cancellation) OR (TI no-show OR AB no-show) OR (TI no-shows OR AB no-shows) OR (TI employment OR AB employment) OR (TI medication OR AB medication) OR (TI death OR AB death) OR (TI deaths OR AB deaths) OR (TI fatality OR AB fatality) OR (TI fatalities OR AB fatalities) OR (MH Mortality+) OR (TI mortality OR AB mortality) OR (TI adherence OR AB adherence) OR (TI nonadherence OR AB nonadherence) | 2774542 |
| 7 | #1 AND #4 AND #5 AND #6 | 1331 |
| 8 | PT (meta-analysis OR “systematic review” OR “literature review” OR meta analysis OR review OR meta-synthesis OR “meta synthesis” ) OR TI (meta-analysis OR “systematic review” OR “literature review” OR “meta analysis” OR meta-synthesis OR “meta synthesis” OR "scoping review" ) | 563763 |
| 9 | #7 NOT #8 | 1258 |
| 10 | #9 AND Narrow by Language: English | 1248 |
| Notes: 3 citations were empty and stated “No Record Available”; Total citations exported: | | 1245 |

**Supplementary Table 4 - PsycInfo (EBSCOhost) – Searched December 2, 2022**

| Search | Query | Results |
| --- | --- | --- |
| 1 | DE "Neoplasms" OR DE "Benign Neoplasms" OR DE "Breast Neoplasms" OR DE "Endocrine Neoplasms" OR DE "Leukemias" OR DE "Melanoma" OR DE "Metastasis" OR DE "Nervous System Neoplasms" OR DE "Terminal Cancer" OR (TI cancer OR AB cancer) OR (TI cancers OR AB cancers) OR (TI malignancy OR AB malignancy) OR (TI malignancies OR AB malignancies) OR (TI tumor OR AB tumor) OR (TI tumors OR AB tumors) OR (TI tumour OR AB tumour) OR (TI tumours OR AB tumours) OR (TI neoplasm OR AB neoplasm) OR (TI neoplasms OR AB neoplasms) | 89886 |
| 2 | (MH Transportation+) OR (MH "Transportation of Patients+") OR (TI transportation OR AB transportation) OR (TI "distance to care" OR AB "distance to care") OR (TI "travel distance" OR AB "travel distance") | 9163 |
| 3 | (((TI transport OR AB transport) OR (TI transports OR AB transports) OR (TI transported OR AB transported) OR (TI transporting OR AB transporting) OR (MH Travel+) OR (TI travel OR AB travel) OR (TI traveled OR AB traveled) OR (TI traveling OR AB traveling) OR (TI travelled OR AB travelled) OR (TI travelling OR AB travelling) OR (MH "Residence Characteristics+") OR (TI residential OR AB residential) OR (TI neighborhood OR AB neighborhood) OR (TI distance OR AB distance) OR (TI residence OR AB residence) OR (TI rural OR AB rural) OR (MH "Rural Population+") OR (TI rurality OR AB rurality)) AND ((TI patient OR AB patient) OR (TI patients OR AB patients) OR (TI inpatient OR AB inpatient) OR (TI inpatients OR AB inpatients))) | 19318 |
| 4 | #2 OR #3 | 28176 |
| 5 | (TI "financial toxicity" OR AB "financial toxicity") OR (TI "financial stress" OR AB "financial stress") OR (TI "financial distress" OR AB "financial distress") OR (TI "financial burden" OR AB "financial burden") OR (TI "financial hardship" OR AB "financial hardship") OR (TI "financial hardships" OR AB "financial hardships") OR (TI "health care cost" OR AB "health care cost") OR (TI "health care costs" OR AB "health care costs") OR (TI "healthcare cost" OR AB "healthcare cost") OR (TI "healthcare costs" OR AB "healthcare costs") OR (TI expenditure OR AB expenditure) OR (TI expenditures OR AB expenditures) OR (TI income OR AB income) OR (TI "drug cost" OR AB "drug cost") OR (TI "drug costs" OR AB "drug costs") OR (TI cost OR AB cost) OR (TI costs OR AB costs) OR (TI finances OR AB finances) OR (TI financial OR AB financial) OR (TI "cost of illness" OR AB "cost of illness") OR (TI "insurance coverage" OR AB "insurance coverage") OR (TI out-of-pocket OR AB out-of-pocket) OR (TI "out of pocket" OR AB "out of pocket") OR (TI "insurance premium" OR AB "insurance premium") OR (TI "insurance premiums" OR AB "insurance premiums") OR (TI "insurance cost" OR AB "insurance cost") OR (TI "insurance costs" OR AB "insurance costs") OR (TI affordable OR AB affordable) OR (TI affordability OR AB affordability) | 238262 |
| 6 | (TI outcome OR AB outcome) OR (TI outcomes OR AB outcomes) OR (TI survival OR AB survival) OR (DE "Quality of Life" OR DE "Health Related Quality of Life" OR DE "Quality of Work Life") OR (TI "quality of life" OR AB "quality of life") OR (TI quality-of-life OR AB quality-of-life) OR (DE "Hospitalization" OR DE "Hospital Admission" OR DE "Hospital Discharge" OR DE "Hospitalized Patients") OR (TI hospitalization OR AB hospitalization) OR (TI hospitalizations OR AB hospitalizations) OR (TI emergency OR AB emergency) OR (TI emergencies OR AB emergencies) OR (TI screening OR AB screening) OR (TI diagnose OR AB diagnose) OR (TI diagnosis OR AB diagnosis) OR (TI diagnoses OR AB diagnoses) OR (TI treatment OR AB treatment) OR (TI treatments OR AB treatments) OR (TI therapy OR AB therapy) OR (TI therapies OR AB therapies) OR (TI chemotherapy OR AB chemotherapy) OR (TI chemotherapies OR AB chemotherapies) OR (TI radiation OR AB radiation) OR (TI appointment OR AB appointment) OR (TI appointments OR AB appointments) OR (TI discontinue OR AB discontinue) OR (TI discontinuation OR AB discontinuation) OR (TI delay OR AB delay) OR (TI delays OR AB delays) OR (TI cancel OR AB cancel) OR (TI cancels OR AB cancels) OR (TI cancellation OR AB cancellation) OR (TI no-show OR AB no-show) OR (TI no-shows OR AB no-shows) OR (TI employment OR AB employment) OR (TI medication OR AB medication) OR (TI death OR AB death) OR (TI deaths OR AB deaths) OR (TI fatality OR AB fatality) OR (TI fatalities OR AB fatalities) OR (DE "Death and Dying" OR DE "Mortality Rate" OR DE "Mortality Risk") OR (TI mortality OR AB mortality) OR (TI adherence OR AB adherence) OR (TI nonadherence OR AB nonadherence) | 1610707 |
| 7 | #1 AND #4 AND #5 AND #6 | 247 |
| 8 | TI (meta-analysis OR “systematic review” OR “literature review” OR “meta analysis” OR meta-synthesis OR “meta synthesis” OR "scoping review" ) | 56034 |
| 9 | #7 NOT #8 | 241 |
| 10 | #9 AND Narrow by Language: English | 239 |

**Supplementary Table 5 - Healthcare Administration (ProQuest): Searched December 2, 2022**

| Search | Query | Results |
| --- | --- | --- |
| 1 | noft((cancer OR cancers OR malignancy OR malignancies OR tumor OR tumors OR tumour OR tumours OR neoplasm OR neoplasms)) | 223049 |
| 2 | noft((transportation OR "distance to care" OR "travel distance" OR ((transport OR transports OR transported OR transporting OR travel OR traveled OR traveling OR travelled OR travelling OR residential OR neighborhood OR distance OR residence OR rural OR rurality) AND (patient OR patients OR inpatient OR inpatients)))) | 124873 |
| 3 | noft("financial toxicity" OR "financial stress" OR "financial distress" OR "financial burden" OR "financial hardship" OR "financial hardships" OR "health care cost" OR "health care costs" OR "healthcare cost" OR "healthcare costs" OR expenditure OR expenditures OR income OR "drug cost" OR "drug costs" OR cost OR costs OR finances OR financial OR "cost of illness" OR "insurance coverage" OR out-of-pocket OR "out of pocket" OR "insurance premium" OR "insurance premiums" OR "insurance cost" OR "insurance costs" OR affordable OR affordability) | 1202118 |
| 4 | noft(outcome OR outcomes OR survival OR "quality of life" OR quality-of-life OR hospitalization OR hospitalizations OR emergency OR emergencies OR screening OR diagnose OR diagnosis OR diagnoses OR treatment OR treatments OR therapy OR therapies OR chemotherapy OR chemotherapies OR radiation OR appointment OR appointments OR discontinue OR discontinuation OR delay OR delays OR cancel OR cancels OR cancellation OR no-show OR no-shows OR employment OR medication OR death OR deaths OR fatality OR fatalities OR mortality OR adherence OR nonadherence) | 1356034 |
| 5 | #1 AND #2 AND #3 AND #4 | 422 |
| 6 | title(("meta analysis" OR "systematic review" OR systematic-review OR meta-analysis OR scoping-review OR literature-review)) | 30485 |
| 7 | #5 NOT #6 | 418 |
| 8 | #7 AND Limit to: English language | 417 |
